# Development of TaqMan-based real-time RT-PCR assay based on *N* gene for the quantitative detection of feline morbillivirus

**DOI:** 10.1186/s12917-021-02837-6

**Published:** 2021-03-23

**Authors:** Siti Tasnim Makhtar, Sheau Wei Tan, Nur Amalina Nasruddin, Nor Azlina Abdul Aziz, Abdul Rahman Omar, Farina Mustaffa-Kamal

**Affiliations:** 1 Faculty of Veterinary Medicine, Universiti Putra Malaysia, UPM Serdang, Selangor, Malaysia; 2 Institute of Bioscience, Universiti Putra Malaysia, UPM Serdang, Selangor, Malaysia

**Keywords:** Feline morbillivirus, TaqMan-based real-time RT-PCR, *N* gene

## Abstract

**Background:**

*Morbilliviruses* are categorized under the family of *Paramyxoviridae* and have been associated with severe diseases, such as Peste des petits ruminants, canine distemper and measles with evidence of high morbidity and/or could cause major economic loss in production of livestock animals, such as goats and sheep. Feline morbillivirus (FeMV) is one of the members of *Morbilliviruses* that has been speculated to cause chronic kidney disease in cats even though a definite relationship is still unclear. To date, FeMV has been detected in several continents, such as Asia (Japan, China, Thailand, Malaysia), Europe (Italy, German, Turkey), Africa (South Africa), and South and North America (Brazil, Unites States). This study aims to develop a TaqMan real-time RT-PCR (qRT-PCR) assay targeting the *N* gene of FeMV in clinical samples to detect early phase of FeMV infection.

**Results:**

A specific assay was developed, since no amplification was observed in viral strains from the same family of *Paramyxoviridae*, such as canine distemper virus (CDV), Newcastle disease virus (NDV), and measles virus (MeV), and other feline viruses, such as feline coronavirus (FCoV) and feline leukemia virus (FeLV). The lower detection limit of the assay was 1.74 × 10^4^ copies/μL with Cq value of 34.32 ± 0.5 based on the cRNA copy number. The coefficient of variations (CV) values calculated for both intra- and inter-assay were low, ranging from 0.34–0.53% and 1.38–2.03%, respectively. In addition, the clinical sample evaluation using this assay showed a higher detection rate, with 25 (35.2%) clinical samples being FeMV-positive compared to 11 (15.5%) using conventional RT-PCR, proving a more sensitive assay compared to the conventional RT-PCR.

**Conclusions:**

The TaqMan-based real-time RT-PCR assay targeting the *N* gene described in this study is more sensitive, specific, rapid, and reproducible compared to the conventional RT-PCR assay targeting the *N* gene, which could be used to detect early infection in cats.

## Background

Family *Paramyxoviridae* belongs to the order *Mononegavirales* and can be further classified into four subfamilies: *Rubulavirinae*, *Avulavirinae*, *Metaparamyxovirinae*, and subfamily *Orthoparamyxovirinae* [1]. Feline morbillivirus (FeMV) is a novel virus under the subfamily *Orthoparamyxovirinae*, which has been associated with the occurrence of chronic kidney disease in cats. FeMV was first discovered in Hong Kong in 2012, followed by that in other countries such as Japan, United States, Italy, Turkey, Thailand, Germany, and Brazil [2–8]. Similar to other morbilliviruses, the FeMV genome is composed of six genes (3′-*N*-*P*/*V*/*C*-*M*-*F*-*H*-*L*-5′), which encode for six structural and two non-structural proteins [8]. The RNA of the virus is being encapsidated by the nucleoprotein (*N*), which makes up for the nucleocapsid [9]. The large protein (*L*) and phosphoprotein (*P*) form the RNA-dependent RNA polymerase (RdRp) complex that is responsible for all polymerase activities, which only recognized the encapsidated RNA as a template [10]. Fusion (*F*) and hemagglutinin (*H*) proteins are glycoprotein which form the envelope of the virus [1]. The matrix (*M*) protein is a non-glycosylated, hydrophobic viral protein, which acts as a bridge to connect the envelope protein with ribonucleoprotein (RNP) core consisting the nucleocapsid, *L* protein, and *P* protein [11]. In relation to that, a putative FeMV recombination between a Japan isolate (ChJ073) with Hong Kong isolate (776 U) involving fusion (*F*) and hemagglutinin (*H*) genes has been documented for a Japanese strain (MiJP003) [12]. This type of recombination may occur when two different virus strains infect the same cats, which also raise a speculation of the possibility that the FeMV strain closely related to 776 U may also be circulating in Japan.

Since the discovery of this novel virus, a number of publications describe utilizing conventional PCR assays to screen FeMV [6–8, 13]. However, the screening of FeMV by conventional PCR assays can be hampered by the low quantity of RNA in the samples [13, 14]. Furthermore, screening large number of samples and performing quantitative analysis are either laborious or impossible to be performed with conventional PCR assays. A previous study has detected 82 FeMV-positive cats out of 208 cats in Malaysia via conventional PCR [15]. Furthermore, a high range of 85–99% nucleotide similarities were obtained from the analysis of partial nucleocapsid (*N*) gene from these FeMV-positive cats. To the best of the author’s knowledge, there were only two published studies describing the development of quantitative-based assay to detect FeMV targeting the *L* and *P*/*V*/*C* gene [14, 16].

As the genetic diversity of FeMV is evidenced in other Asian countries such as Japan, hence, there is a need for a robust diagnostic molecular tool to detect the genetically heterogenous viral strains [17]. This research aims to develop TaqMan real-time RT-PCR (qRT-PCR) assay based on the *N* gene. *N* gene is an ideal target gene because it is a well-conserved gene and a major viral protein that folds and protects the viral RNA, playing a key role in virus replication [18]. The *N* gene sequence was used to design the primers and probe sequences to develop TaqMan-based real-time PCR assay. Both specificity and sensitivity of the assay were assessed and further evaluated with clinical samples alongside the conventional RT-PCR. Ultimately, developing this specific and sensitive assay for FeMV will galvanize the subsequent detailed examination on the significance of this virus in domestic cat populations.

## Results

### Optimization of the TaqMan-based real-time RT-PCR (qRT-PCR) targeting *N* gene of FeMV

The TaqMan-based qRT-PCR was optimized by determining the optimal primers and probe concentration, in which 0.8 μL of 10 μM primers and 0.2 μL of 10 μM probe were used.

### Sensitivity of TaqMan-based real-time RT-PCR (qRT-PCR) targeting *N* gene of FeMV

**Fig. 1:**
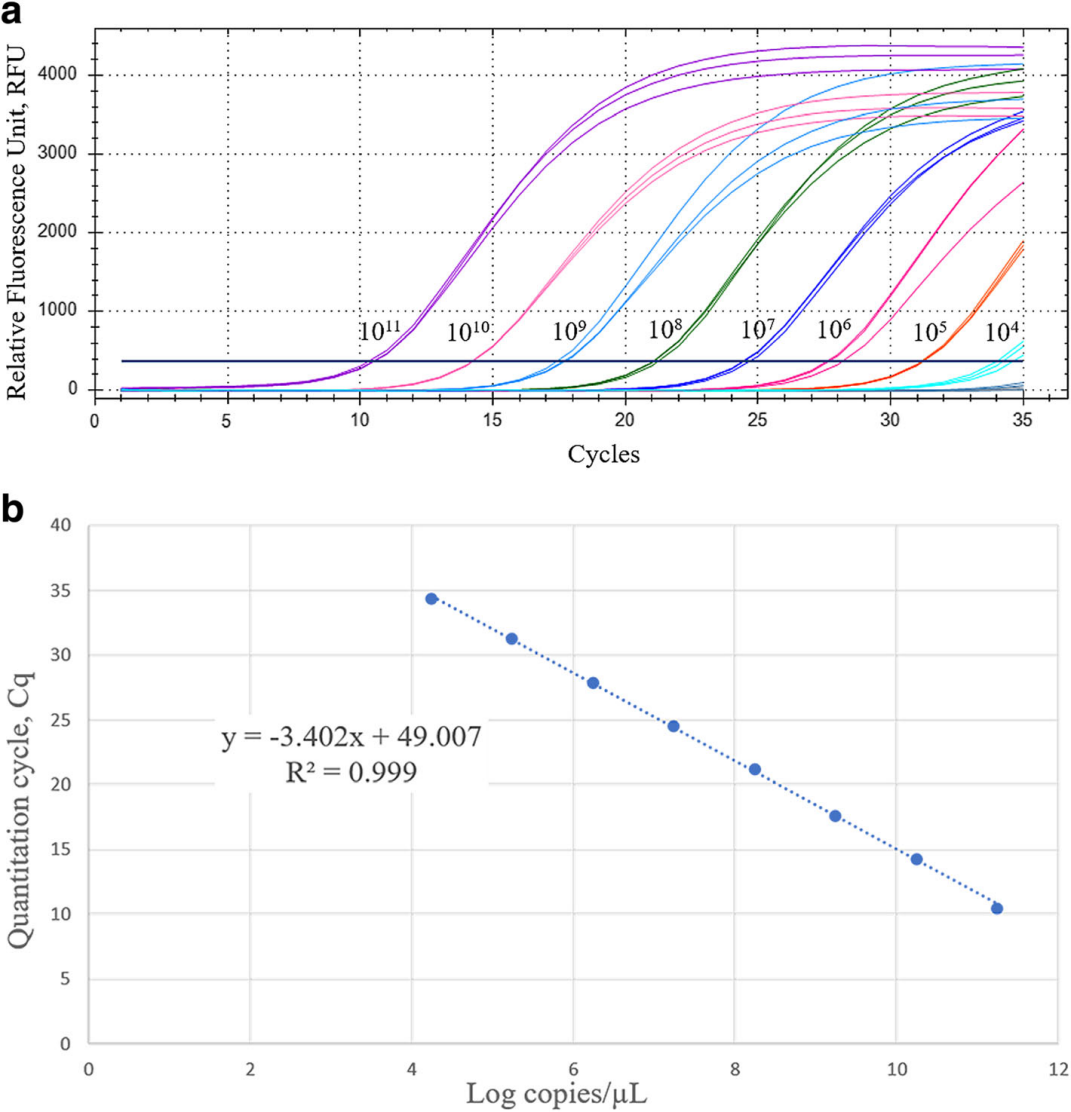
**a** The detection limit of qRT-PCR assay based on cRNA copy number. Amplification plot of 10-fold serial diluted cRNA ranging from 1.74 × 10^11^ to 1.74 × 10^4^ copies/μL. **b** Standard curve of qRT-PCR assay. Ten-fold dilutions of control cRNA were assessed with the qRT-PCR assay. Log copies per μL and quantitation cycle (Cq) are plotted on the x-axis and y-axis, respectively

The concentration of the cRNA was 12 ng/μL, which was equivalent to 1.74 × 10^11^ copies/μL. A series of 10-fold dilution of the cRNA ranging from 1.74 × 10^1^ to 1.74 × 10^11^ was reverse transcribed and subsequently used to determine the sensitivity of the real-time PCR assay. The lower detection limit based on the cRNA copy number was achieved at 1.74 × 10^4^ copies/μL, with a corresponding Cq value of 34.32 ± 0.5 (Fig. 1a). The standard curve of the qRT-PCR assay based on the cRNA copy number was plotted in linear with the coefficient of determination (R^2^) of 0.999, the slope of − 3.402 and the efficiency of 96.8% (Fig. 1b).

### Specificity of TaqMan-based real-time RT-PCR (qRT-PCR) targeting N gene of FeMV

**Fig. 2:**
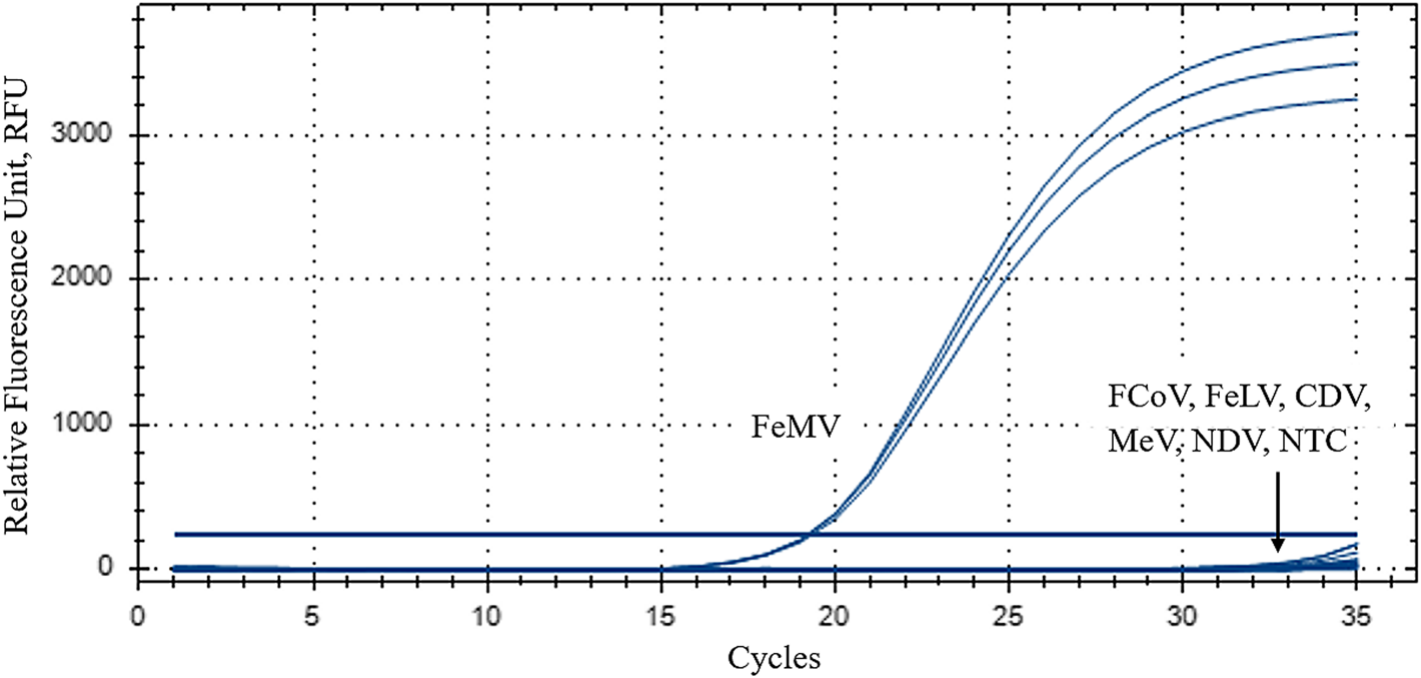
Specificity of *N* gene based on the qRT-PCR assay. Amplification plot detection of FeMV, FCoV, FeLV, CDV, MeV, NDV, and no-template control (NTC). Amplification curve was only detected for FeMV (positive control)

Specificity of the qRT-PCR assay was assessed against the viral strains from the same family *Paramyxoviridae*, which were the Newcastle disease virus (NDV), canine distemper virus (CDV), measles virus (MeV), and other feline viruses, such as feline leukemia virus (FeLV) and feline coronavirus (FCoV). None of the RNA viruses showed amplification signals, proving the high specificity of the assay (Fig. 2). Besides that, the no-template control did not show any amplification signal.

### Reproducibility of TaqMan-based real-time RT-PCR (qRT-PCR) targeting the *N* gene of FeMV

The reproducibility of the qRT-PCR was determined by the intra- and inter-assay evaluation of the Cq value using RNAs from three different positive samples: UPM23, UPM52, and UPM202. The intra-assay was analyzed by using the RNAs from these three samples in triplicates per run; whereas for the inter-assay assessment, the RNAs were amplified in triplicates in three different consecutive runs. The calculated SD and CV values for the intra-assay variations ranged from 0.10 to 0.17 and from 0.34 to 0.53%, respectively (Table 1). Meanwhile, for the inter-assay evaluation, the calculated SD and CV values ranged from 0.45 to 0.66 and 1.38 to 2.03%, respectively (Table 2).

**Table 1:** Intra-assay variation

Sample	Cq value			Mean Cq	S.D.	CV%
	Replicate 1	Replicate 2	Replicate 3			
UPM23	28.00	28.01	27.83	27.95	0.1	0.36
UPM52	32.92	32.77	32.71	32.80	0.11	0.34
UPM202	32.17	32.26	32.49	32.31	0.17	0.53

**Table 2:** Inter-assay variation

Sample	Cq value			Mean Cq	S.D.	CV%
	Assay 1	Assay 2	Assay 3			
UPM23	27.42	27.66	28.42	27.83	0.52	1.87
UPM52	32.33	32.57	33.20	32.70	0.45	1.38
UPM202	31.90	32.44	33.21	32.51	0.66	2.03

### Clinical samples evaluation using TaqMan-based qRT-PCR and comparison with conventional RT-PCR assay

Urine samples (*n* = 55) and kidney samples (*n* = 16) were evaluated to determine the performance of the newly developed qRT-PCR assay (Table 3). Among these 71 samples, 22/55 (40.0%) urine and 3/16 (18.8%) kidney samples were positive for FeMV with log copy number ranging from 3.54 ± 0.01 to 6.60 ± 0.12. Besides that, among the 16 cats that had both kidney and urine samples collected, none of these cats had detectable FeMV RNA in both samples concurrently. For example, the three cats showing FeMV-positive kidney samples (UPM304, UPM312, and UPM321) had no detectable FeMV RNA in their urine samples. Conversely, one of the cats (UPM305) that showed detectable FeMV RNA in its urine sample had no FeMV RNA in its kidney sample. In order to determine the sensitivity of TaqMan qRT-PCR assay in detecting FeMV from the clinical samples, conventional RT-PCR using two different sets of published primers targeting *N* gene [12] was conducted on all 71 samples, in which only 11/55 (20%) urine samples were positive, while no (0/16; 0%) kidney samples were detected positive.

**Table 3:** Detection of FeMV using conventional RT-PCR and qRT-PCR quantification from clinical samples

Sample	Sample ID	RT-PCR (***N*** gene)	Log copy number (mean ± S.D.)	Source of cat	Health status
Urine	UPM23	+	6.60 ± 0.12	Client-owned	Healthy
Urine	UPM52	+	4.94 ± 0.02	Client-owned	Healthy
Urine	UPM53	+	4.93 ± 0.02	Client-owned	Healthy
Urine	UPM202	+	4.77 ± 0.02	Client-owned	Healthy
Urine	UPM203	–	BLD	Client-owned	Healthy
Urine	UPM204	–	4.86 ± 0.06	Client-owned	Healthy
Urine	UPM205	–	4.55 ± 0.03	Client-owned	Healthy
Urine	UPM207	–	4.67 ± 0.02	Client-owned	Healthy
Urine	UPM210	+	3.66 ± 0.08	Client-owned	Healthy
Urine	UPM219	–	3.80 ± 0.14	Client-owned	CKD stage IV
Urine	UPM220	+	4.11 ± 0.05	Client-owned	N/A
Urine	UPM221	–	3.66 ± 0.02	Client-owned	Healthy
Urine	UPM224	–	BLD	Client-owned	Healthy
Urine	UPM225	–	3.54 ± 0.01	Client-owned	Healthy
Urine	UPM226	–	3.71 ± 0.11	Client-owned	Healthy
Urine	UPM227	–	BLD	Client-owned	N/A
Urine	UPM228	–	BLD	Client-owned	N/A
Urine	UPM229	–	BLD	Client-owned	N/A
Urine	UPM231	+	4.29 ± 0.23	Client-owned	Healthy
Urine	UPM232	–	BLD	Client-owned	CKD Stage II
Urine	UPM240	–	BLD	Client-owned	Healthy
Urine	UPM241	–	BLD	Client-owned	Healthy
Urine	UPM271	–	BLD	Client-owned	Heart disease
Urine	UPM272	–	BLD	Client-owned	CKD Stage II
Urine	UPM273	–	BLD	Client-owned	N/A
Urine	UPM274	–	BLD	Client-owned	Suspected CKD
Urine	UPM275	–	BLD	Client-owned	FURD
Urine	UPM276	+	4.05 ± 0.08	Client-owned	Healthy
Urine	UPM277	–	BLD	Client-owned	Healthy
Urine	UPM278	–	BLD	Client-owned	N/A
Urine	UPM279	–	BLD	Client-owned	N/A
Urine	UPM280	–	BLD	Client-owned	Suspected CKD
Urine	UPM281	–	BLD	Client-owned	N/A
Urine	UPM282	–	4.63 ± 0.05	Client-owned	FLUTD
Urine	UPM283	–	BLD	Client-owned	Healthy
Urine	UPM284	–	BLD	Client-owned	Healthy
Urine	UPM285	–	3.74 ± 0.02	Client-owned	N/A
Urine	UPM286	–	3.88 ± 0.18	Client-owned	Obstructive FLUTD
Urine	UPM287	–	BLD	Client-owned	Suspected CKD
Urine	UPM304	–	BLD	Shelter	N/A
Urine	UPM305	+	4.34 ± 0.10	Shelter	*Platynosomum* sp.
Urine	UPM306	–	3.76 ± 0.26	Shelter	N/A
Urine	UPM307	–	BLD	Shelter	N/A
Urine	UPM308	–	BLD	Shelter	*Platynosomum* sp.
Urine	UPM310	–	BLD	Shelter	N/A
Urine	UPM311	–	BLD	Shelter	*Platynosomum* sp.
Urine	UPM312	–	BLD	Shelter	*Platynosomum* sp.
Urine	UPM313	–	BLD	Shelter	N/A
Urine	UPM314	+	4.45 ± 0.07	Shelter	*Platynosomum* sp.
Urine	UPM315	+	4.92 ± 0.07	Shelter	*Platynosomum* sp.
Urine	UPM316	–	BLD	Shelter	*Platynosomum* sp.
Urine	UPM317	–	BLD	Shelter	N/A
Urine	UPM318	–	BLD	Shelter	N/A
Urine	UPM320	–	BLD	Shelter	N/A
Urine	UPM321	–	BLD	Shelter	N/A
Kidney	UPM304	–	3.80 ± 0.03	Shelter	N/A
Kidney	UPM305	–	BLD	Shelter	*Platynosomum* sp.
Kidney	UPM306	–	BLD	Shelter	N/A
Kidney	UPM307	–	BLD	Shelter	N/A
Kidney	UPM308	–	BLD	Shelter	*Platynosomum* sp.
Kidney	UPM310	–	BLD	Shelter	N/A
Kidney	UPM311	–	BLD	Shelter	*Platynosomum* sp.
Kidney	UPM312	–	3.70 ± 0.10	Shelter	*Platynosomum* sp.
Kidney	UPM313	–	BLD	Shelter	N/A
Kidney	UPM314	–	BLD	Shelter	*Platynosomum* sp.
Kidney	UPM315	–	BLD	Shelter	*Platynosomum* sp.
Kidney	UPM316	–	BLD	Shelter	*Platynosomum* sp.
Kidney	UPM317	–	BLD	Shelter	N/A
Kidney	UPM318	–	BLD	Shelter	N/A
Kidney	UPM320	–	BLD	Shelter	N/A
Kidney	UPM321	–	3.89 ± 0.08	Shelter	N/A

*BLD* denotes below limit of detection of qRT-PCR; and

*N/A* denotes not available

Interestingly, the cats that were deemed healthy with their health status available had a higher positive rate of detection (13/20) compared to the cats diagnosed with a disease (3/10). The source of which the cats was collected from, be it from the shelters or were originally pet cats, may also be an important factor in the FeMV-positive detection rate. A higher percentage of positive detection rate was observed among client-owned cats (46%; 18/39) compared to the shelter cats (43%; 7/16).

Biological samples taken from the cats in shelters were also part of another study that assessed the presence of *Platynosomum* sp., a hepatic trematode found in the gall bladder and biliary ducts of cats. Incidentally, *Platynosomum* sp. was detected in 7 out of 16 (43.8%) shelter cats, of which 4 (57.1%) of them were also FeMV-positive.

## Discussion

Feline morbillivirus (FeMV) has been speculated to cause chronic kidney disease (CKD) and it is commonly detected in urine and kidney tissue samples. Previously, conventional assays, such as RT-PCR and reverse transcription loop-mediated isothermal amplification (RT-LAMP), have been developed for targeting the *L* gene of FeMV [5, 7, 8, 19, 20]. However, these conventional assays were qualitative and required additional step of gel electrophoresis to visualize the PCR product. In this study, a two-step TaqMan-based qRT-PCR diagnostic assay for the detection of FeMV-*N* gene was developed. This quantitative assay was designed to detect the *N* gene, one of the most conserved regions in morbilliviruses. Other quantitative-based assays, specifically qRT-PCR, has also been developed to detect FeMV, but all were designed to either target the *L* gene or the *P*/*V*/*C* gene [14, 16]. Moreover, these qRT-PCR assays had low positive rate of detection, which could be due to the limited availability of FeMV sequences at the time, thereby resulting in a less inclusive assay. Hence, it is crucial to design the primers and probe based on the latest sequences available. In addition, *P* gene has been shown to have the highest rates of nucleotide polymorphisms, ranging from 88.6 to 99.3% [12]. Besides that, a study conducted on *peste des petits ruminants virus* (PPRV), another morbillivirus member, revealed that the *L* gene had the highest evolution rate at 9.75 × 10^− 4^ site per year, while the *N* gene showed the lowest evolution rate at 1.1 × 10^− 3^ site per year [21].

Nucleoprotein (*N*) comprises two regions: core domain (*N*_CORE_) and C-terminal domain (also known as *N*_TAIL_) [22]. *N*_CORE_ covers the first 400 amino acids of the *N* protein and it is responsible for the RNA encapsidation prior to viral replication and translation. This core domain also consists of three previously identified conserved motifs. *N* protein also possesses nuclear export signal, nuclear localization signal (NLS), and RNA binding motifs. Both nuclear export signal and NLS are believed to transport the *N* protein to the nucleus of the host cell, while the RNA binding site is considered to be involved in *N*-*N* self-interaction and interaction of *N*-*N* RNA monomers during genomic RNA binding. *N*-_TAIL_ region comprises structurally variable region of approximately 120 to 150 amino acids, which mediate the interaction with *P* gene [23]. Besides that, *N* gene is integral during the initial infection, as the RNA synthesis by RdRp complex will only recognize and synthesize the viral RNA encapsidated by *N* protein as a template [10]. Given that the *N* gene is conserved compared to other genes of FeMV and detectable during the early phase of infection, a quantitative assay targeting the *N* gene of FeMV was developed in this study. In order to develop this assay, a partial sequence of ~ 1.5 kb *N* gene was obtained from seven local isolates. Upon obtaining the ~ 1.5 kb of FeMV-*N* gene sequences of these isolates, the primers and probe for TaqMan-based qRT-PCR assay were designed specifically to target 122 bp of *N* gene.

For the development of TaqMan-based real-time RT-PCR (qRT-PCR), a standard curve was generated to accurately quantify the FeMV. The sensitivity of the developed assay was done by quantifying 10-fold serial dilutions of standard complementary RNA (cRNA). The results showed that the assay could detect up to 10,000 copy number of FeMV. Even though the resulting sensitivity of this newly developed qRT-PCR assay was low, this assay has done none of the other established assays had to date, which was utilizing newly designed primers and probe set targeting *N* gene. The specificity test of the assay was performed by running the assay against other viral strains from the same family, which were NDV, CDV, MeV, and two feline viruses: FeLV and FCoV. The absence of cross-reaction proved high specificity of the developed assay. In addition to that, the low sensitivity reported for this assay could be due to the highly specific designed primers based on stringent parameters, as evident by the absence of amplification in other tested viruses. In a diagnostic test, a highly specific assay may compromise the sensitivity of the assay, resulting in an inverse relationship [24]. Furthermore, incorporating probe known for its specificity in this assay might also contribute to a highly specific qRT-PCR assay, thus compromising its sensitivity. In order to evaluate the reproducibility of the assay, intra- and inter-assay assessments were done by testing three different positive samples: UPM23, UPM52, and UPM202. The value of standard deviation calculated for both intra- and inter-assay indicated that the developed qRT-PCR assay was low in variability but high in reproducibility. Besides that, comparing the coefficient of variation (CV) of both intra- and inter-assay with a previously reported study indicated that the value of CV recorded in this work was relatively low, confirming the reproducibility of this assay [16].

In order to further evaluate the sensitivity of the qRT-PCR assay, clinical samples (*n* = 71) were tested to detect FeMV by targeting the *N* gene and compared with conventional RT-PCR assay. All samples that were detected positive by conventional RT-PCR (11/71) were also detected by the qRT-PCR assay. However, there were 15 qRT-PCR FeMV-positive samples that were not detected via conventional RT-PCR. Some of these samples had a copy number of ~ 1000. Based on the standard curve, even though the lowest result of limit of detection was ~ 10,000 copy number, the quantification on clinical samples proved that the newly designed qRT-PCR assay was able to amplify samples which had a lower copy number.

Evaluation of the clinical samples showed that healthy cats had a higher positive rate compared to cats diagnosed with a disease. Since this research was a cross-sectional study, it was difficult to interpret whether or not this finding was significant. Nevertheless, a prospective follow-up of these cats may provide an insight on the clinical relationship of the cats with FeMV infection. Besides that, a higher rate of detection was observed among client-owned cats compared to shelter cats, which was consistent with a previous study although it was contradictory to other studies [2, 15]. This observation could be due to the differences in the shelter management among different countries. Shelter cats are usually placed in an enclosed area, limiting their interactions with free-roaming cats, hence, reducing the transmission from one cat to another. Furthermore, the transmission among client-owned cats may occur if the cats are living outdoors or if they are semi-roamer cats, as they can interact with other free-roaming cats [25]. Additionally, a multi-cat household may have a higher chance to transmit the virus to other healthy cats already living within the household or newly introduced animals [3]. It was also noted that all cats were not detected positive for both urine and kidney samples, as observed by many studies as well [4, 26]. This observation may be caused by the early-stage infection, whereby the virus has yet to travel to the kidney. Consequently, this finding would suggest for a further study to observe the viral shedding and specifically, the viral load over time, accompanied with serum urea and creatinine level among FeMV-positive cats to determine the relationship of the severity of kidney disease and FeMV pathogenesis.

The cats recruited in this study were also part of a study to detect the presence of *Platynosomum* sp., a fluke living in pancreatic ducts, bile ducts, and gall bladder [27]. This parasite requires two intermediate hosts, a snail, followed by a second intermediate host such as skink, gecko, lizard, or toad. A cat can be infected with *Platynosomum* sp. after ingesting an infected second intermediate host. The clinical signs of *Platynosomum* sp. infection vary from asymptomatic to progressive disease, and it can also cause death due to hepatic failure and biliary tract obstruction. Biological samples of the liver, bile ducts, and feces were obtained from the shelter cats in this study, in which *Platynosomum* sp. was detected in 4 out of 7 FeMV-positive cats. Although this finding was incidental, co-infection of FeMV and *Platynosomum* sp. should be further explored, especially in shelter cat populations, given that this cohort of cats could potentially harbor many pathogens.

The developed qRT-PCR assay can be utilized for field samples given its high specificity and novelty of targeting the *N* gene of FeMV. Although there has yet a clear indication on whether or not this virus is involved in the pathogenesis of chronic kidney disease in cats, further studies are needed to determine the extent of infection among cats and its clinical significance.

## Conclusion

In conclusion, a probe-based qRT-PCR assay targeting the *N* gene of FeMV has been successfully optimized and validated. This study is the first quantitative assay targeting the FeMV-*N* gene, which is integral in RNA synthesis by RdRp complex. Even though the developed assay demonstrated a lower sensitivity compared to the previously designed qRT-PCR assay targeting *L* and *P*/*V*/*C* gene; in comparison to the conventional RT-PCR, it can detect samples with low viral load. Hence, the developed qRT-PCR assay can be applied in diagnostic and quantification of the FeMV viral load, especially during the early stage of infection.

## Materials and methods

### Total RNA extraction and cDNA synthesis

The total RNA extraction for collected samples was performed by using Direct-zol™ RNA MiniPrep Plus kit (Zymo Research, California, United States) following the manufacturer’s guidelines. Extracted RNA was first subjected to cDNA synthesis prior to conventional RT-PCR and qRT-PCR assays. cDNA synthesis was performed by using the SensiFAST™ cDNA Synthesis Kit (Bioline, London, UK) with 20 μL reaction consisting of 10 μL purified RNA, 4 μL of 5x TransAmp Buffer, 1 μL of reverse transcriptase, and 5 μL of nuclease-free water. The reaction was carried out at 25 °C for 10 min, 42 °C for 15 min, 48 °C for 15 min, and 85 °C for 5 min. The cDNA was stored at − 20 °C until use.

### TaqMan-based real-time RT-PCR (qRT-PCR) assay

Primer set and probe were designed based on partial *N* gene sequences of FeMV-Malaysia isolates (UPM23, UPM52, UPM53, UPM210, UPM231, UPM305, and UPM315) using Integrated DNA Technologies (IDT) software (Table 4). The designed probe and primer set were then compared with the alignment of closely related FeMV from other countries’ isolates: Japan (SS3, MiJP003, ChJP073), China (M252A), and Thailand (Thai-U16) (Table 4). Specific sequences for primers and probe used to yield 122 bp PCR amplicons are as follows: forward primer, GGTCAAGAGATGGTGAGAAGAT; reverse primer, CCAGATTCACCTCCCGAATTA; and probe, TTTGCGCGAGAACTTGGGCTATCT. The FeMV TaqMan probe was labeled with 6-FAM at 5′-end, and fluorescence quencher, ZEN and IBFQ at an internal site and 3′-end, respectively. Real-time RT PCR was performed using CFX96 machine (Bio-Rad, California, USA) utilizing SensiFAST™ Probe NO-ROX Kit (Bioline, London, UK) following the manufacturer’s suggestion. Briefly, each reaction well consisted of 4 μL cDNA, 10 μL of 2x SensiFAST Probe No-ROX Mix, a final concentration of 400 nM for each forward and reverse FeMV primers, 100 nM probe, and nuclease-free water up to the final volume of 20 μL. The assay was performed at 95 °C for 2 min, followed by 35 cycles at 95 °C for 15 s and 54 °C for 45 s, where each sample was run in triplicates.

**Table 4:** GenBank accession number

Isolate	Accession Number
SS3	LC036587
MiJP003	AB924121
ChJP073	AB924122
M252A	JQ411016
Thai-U16	MF627832
UPM23	MN264638
UPM52	MN264639
UPM53	MN264640
UPM210	MN264641
UPM231	MN264642
UPM305	MN792827
UPM315	MN792828

### Standard RNA preparation

Generation of standard RNA was prepared by subcloning the partial sequence of ~ 1.5 kb FeMV-*N* gene into a pSC-A-amp/kan plasmid vector by using StrataClone PCR cloning kit (Agilent Technologies, California, United States) according to the manufacturer’s instruction. After linearization, RNA transcription was performed using the Riboprobe® in vitro Transcription Systems (Promega, Wisconsin, United States) with the T7 RNA polymerase promoter site available in vector pSC-A-amp/kan following the manufacturer’s direction. After DNase treatment, the transcript was purified by phenol-chloroform purification and quantified by using spectrophotometer (Bio-Rad, California, USA). The copy number of cRNA calculated was 1.74 × 10^11^ copies/μL. cRNA was diluted in 10-fold serial dilution and converted into cDNA prior to standard curve generation.

### Specificity and sensitivity

The specificity of the developed assay was assessed by testing on viral RNA from same genus, which were canine distemper virus (CDV) and measles virus (MeV), from the same family, Newcastle disease virus (NDV), and from other feline viruses, which were feline leukemia virus (FeLV) and feline coronavirus (FCoV) (Table 5).

**Table 5:** List of viruses used in this study and its sources

Virus	Source
Canine distemper virus (CDV)	Nobivac Puppy DP, Intervet
Measles virus (MeV)	Serum Institute of India LTD, Pune
Newcastle disease virus (NDV)	Department of Vet. Pathology and Microbiology, UPM
Feline leukemia virus (FeLV)	Department of Vet. Pathology and Microbiology, UPM
Feline coronavirus (FCoV)	Department of Vet. Pathology and Microbiology, UPM

The sensitivity of qRT-PCR assay was assessed by running 10-fold serial dilution of cRNA standard (from 1.74 × 10^11^ to 1.74 × 10^2^ copies/μL) to detect the threshold limit of the assay. Ten different 10-fold serial dilutions were performed by adding 1 μL of RNA into 9 μL of nuclease-free water. The mixture was then vortexed for at least 10 s before the same step was repeated for the next dilution. Each of the diluted RNA, along with the stock RNA control, was converted into cDNA and subjected for qRT-PCR in triplicates.

### Reproducibility

In order to assess reproducibility of the developed assay, three different positive samples were selected, which were then subjected for intra- and inter-assay. Three different positive samples (UPM23, UPM52, UPM202) were assayed in the same run in triplicates to evaluate the intra-assay variations. In order to assess for inter-assay variations, the same three samples were subjected for three different consecutive runs in triplicates. Values for mean, standard deviation (SD), and coefficient of variations (CV) for both variation assays were calculated by using Microsoft Excel Software (version 2016, USA).

### Clinical samples collection

Animal ethics application was approved by the Institutional Animal Care and Use Committee (IACUC) of Universiti Putra Malaysia (UPM/IACUC/AUP-R037/2018). Convenient sampling was performed, whereby urine (*n* = 55) and kidney samples (*n* = 16) of cats were collected from veterinary hospital, private veterinary clinics and animal shelters around Klang Valley, Malaysia. The cats presented to the veterinary hospital or private veterinary clinics were either presented for annual health examination, neutering procedure, or due to health-related issues, such as kidney-related and heart diseases. Samples of cats with their serum urea-creatinine data available were further sub-grouped into cats with the presence or absence of kidney-related disease based on the International Renal Interest Society (IRIS) Guidelines. The owner’s consent was requested prior to sample collection. When available, kidney samples (*n* = 16) from post-mortem and corresponding urine samples (*n* = 16) were collected from animal shelters.

### Sample processing

A urine sample was collected into a sterile sample collection bottle either by cystocentesis or manual compression. The supernatant of urine was obtained after a centrifugation step at 2320×g for 5 min. Then, the supernatant was mixed with RNAlater® solution (Ambion, Texas, United States) at a ratio of 1:1 and stored at − 20 °C prior to the RNA extraction. During postmortem, the collected kidney samples were immediately transferred into a sterile collection bottle containing RNAlater® solution. For sample processing, kidney tissues of approximately 1 g were cut into small pieces and crushed by using the pestle and mortar along with sterile sand. Phosphate-buffer saline solution (Gibco, Massachusetts, United States) of 1 g/mL was added into the homogenized kidney. Next, the mixture was transferred into a 15-mL tube to be centrifuged at 2320×g for 5 min to remove any large debris and sand. The kidney lysate was then subjected for total RNA extraction.

### Clinical samples evaluation using TaqMan-based qRT-PCR and conventional RT-PCR

Converted cDNA clinical samples were assessed by conventional RT-PCR using published primers (Table 6) with cDNA of UPM52 as a positive control, together with no-template control following a modified protocol (Table 7).

**Table 6:** Primer sequences used to amplify two different regions of *N* gene

Region	Primer	Sequence (5′-3′)	Product size (bp)	Source
**Middle region**	FN-2F	GTTAGCTTAGGATTTGAGAACCC	680 bp	[12]
	FN-2R	CACCATCTCTTGACCAAGTCT		
**End region**	FN-3F	GCTATGGAGTTATGCCATGGG	637 bp	
	FN-3R	GTTGTGAACCTTGAGGTCCTAAG

**Table 7:** PCR protocol applied for two different primer sets of *N* gene

Step	Temperature	Time	Cycle
**Initial denaturation**	95 °C	1 min	1x
**Denaturation**	95 °C	15 s	
**Annealing**	58 °C	1 min	35x
**Extension**	72 °C	1 min	
**Final extension**	72 °C	5 min	1x
**Hold**	12 °C	∞	1x

TaqMan-based qRT-PCR was performed according to the developed protocol described above with each sample ran in triplicates along with NTC and cDNA of positive control (UPM52). Nuclease-free water (Promega, Wisconsin, United States) was used as template in NTC in both conventional RT-PCR and qRT-PCR assays.

### *Platynosomum* sp. detection from postmortem of shelter cats

Liver, bile duct, and feces samples in rectum were collected in the postmortem investigation of shelter cats to detect the presence of *Platynosomum* sp. Liver samples were used to collect adult fluke, while bile duct and feces samples were used to detect ova. In order to allow for activation and collection of mature flukes, liver samples were extracted and immersed in warm water (38 °C–40 °C). Parasitic burden was calculated using the formula described in a previous study by applying the number of adult flukes collected per sample [28]. For ova collection from bile juice and feces samples, any ova isolated from these clinical samples were pipetted into microcentrifuge tubes with normal saline and they were stored in − 20 °C for further analysis. In fecal examination, two different methods were performed to detect fluke eggs: the simple floatation technique and centrifugal fecal sedimentation test in formal-ether solution [29, 30]. Adult fluke and ova were identified based on a previous study [31].

## Data Availability

All data generated or analyzed in this study can be obtained within the tables and figures of the manuscript.

## Abbreviations

qRT-PCR: Quantitative reverse transcription polymerase chain reaction
FeMV: Feline morbillivirus
CDV: Canine distemper virus
NDV: Newcastle disease
MeV: Measles virus
FCoV: Feline coronavirus
FeLV: Feline leukemia virus
RT-PCR: Reverse transcription polymerase chain reaction
*N*: Nucleocapsid protein
*L*: Large protein
*P*: Phosphoprotein
RdRp: RNA-dependent RNA polymerase
*F*: Fusion protein
*H*: Hemagglutinin protein
*M*: Matrix protein
RNP: Ribonucleoprotein
FURD: Feline upper respiratory disease
FLUTD: Feline lower urinary tract disease
IDT: Integrated DNA technologies
IACUC: Institutional animal care and use committee
UVH: University veterinary hospital
IRIS: International renal interest society
